# Race to the Moon or the Bottom? Applications, Performance, and Ethical Considerations of Artificial Intelligence in Prosthodontics and Implant Dentistry

**DOI:** 10.3390/dj13010013

**Published:** 2024-12-27

**Authors:** Amal Alfaraj, Toshiki Nagai, Hawra AlQallaf, Wei-Shao Lin

**Affiliations:** 1 Department of Prosthodontics and Dental Implantology, College of Dentistry, King Faisal University, Al Ahsa 31982, Saudi Arabia; asalfaraj@kfu.edu.sa; 2 Department of Prosthodontics, Indiana University School of Dentistry, Indianapolis, IN 46202, USA; tnagai@iu.edu; 3 Department of Periodontology, Indiana University School of Dentistry, Indianapolis, IN 46202, USA; halqalla@iu.edu

**Keywords:** deep learning, prosthodontics, ethics, responsible AI principles

## Abstract

**Objectives:** This review aims to explore the applications of artificial intelligence (AI) in prosthodontics and implant dentistry, focusing on its performance outcomes and associated ethical concerns. **Materials and Methods:** Following the PRISMA guidelines, a search was conducted across databases such as PubMed, Medline, Web of Science, and Scopus. Studies published between January 2022 and May 2024, in English, were considered. The Population (P) included patients or extracted teeth with AI applications in prosthodontics and implant dentistry; the Intervention (I) was AI-based tools; the Comparison (C) was traditional methods, and the Outcome (O) involved AI performance outcomes and ethical considerations. The Newcastle–Ottawa Scale was used to assess the quality and risk of bias in the studies. **Results:** Out of 3420 initially identified articles, 18 met the inclusion criteria for AI applications in prosthodontics and implant dentistry. The review highlighted AI’s significant role in improving diagnostic accuracy, treatment planning, and prosthesis design. AI models demonstrated high accuracy in classifying dental implants and predicting implant outcomes, although limitations were noted in data diversity and model generalizability. Regarding ethical issues, five studies identified concerns such as data privacy, system bias, and the potential replacement of human roles by AI. While patients generally viewed AI positively, dental professionals expressed hesitancy due to a lack of familiarity and regulatory guidelines, highlighting the need for better education and ethical frameworks. **Conclusions:** AI has the potential to revolutionize prosthodontics and implant dentistry by enhancing treatment accuracy and efficiency. However, there is a pressing need to address ethical issues through comprehensive training and the development of regulatory frameworks. Future research should focus on broadening AI applications and addressing the identified ethical concerns.

## 1. Introduction

Artificial intelligence (AI), one of the fastest-growing technologies, is the science of creating computers and machines that can mimic human cognitive functions and perform various problem-solving tasks [1]. This technology encompasses various subfields such as machine learning (ML), deep learning (DL), artificial neural networks (ANNs), robotics, natural language processing (NLP), and genetic algorithms [1]. In medicine, Gunn in 1976, was the first to investigate its application in diagnosing abdominal pain using computer analysis [2]. Currently, AI applications in medicine demonstrably assist clinicians in diagnosing diseases, making clinical decisions, and predicting treatment outcomes [2].

Similarly, AI’s advent in dentistry is gradually improving diagnostic imaging, treatment planning, and the delivery of quality care for patients. AI applications have the potential to assist dentists in complex diagnostic tasks, enhancing diagnostic accuracy and efficiency and reducing clinician stress. In dentistry, ML and DL algorithms are commonly utilized among various AI subfields [3]. ML focuses on developing data and algorithms to allow computers or machines to learn from data, akin to human learning. ML encompasses different learning approaches: supervised learning (data labeled for desired outcomes), unsupervised learning (data without labels where algorithms find patterns independently), and semi-supervised learning (a blend of labeled and unlabeled data) [4]. Conversely, DL employs convolutional neural networks (CNNs) to identify patterns in large raw data such as images, sounds, and text, facilitating predictions or analyses [4].

Prosthodontics and implant dentistry involve the treatment and fabrication of removable and fixed dental prostheses, including implant placement and prosthetic rehabilitation [5]. AI integration in this field is emerging as a significant tool for designing and fabricating prostheses [6]. Zhang et al. conducted research using DL models to extract marginal lines more accurately, while Lerner et al. developed an AI model to assist in fabricating fixed implant prostheses with monolithic zirconia crowns, detecting subgingival margins of abutments to aid dentists in tooth preparation and maintaining occlusal contacts [7,8]. AI models also support clinicians in treatment planning for implant surgeries based on patients’ clinical and radiographic scan reports [6]. Incorporating AI in treatment planning can potentially reduce dependence on human judgment, thereby minimizing errors and improving treatment accuracy [9].

Clinicians acknowledge that the rapid development and applications of AI in medical and dental care signify a “race to the moon”, highlighting significant progress and innovation. However, focusing solely on technological advancement without addressing key ethical concerns, such as algorithmic bias, fairness, data security, and patient privacy, risks a descent “to the bottom”. In this scenario, these issues may become amplified and the benefits of AI undermined. Ethical challenges, regulatory compliance, and inadequate professional training in AI further complicate its integration into healthcare. Moreover, many developing and low-income countries face barriers to accessing these advanced technologies, contributing to increasing healthcare disparities [10].

This review aims to provide a comprehensive synthesis of the current evidence on AI applications in prosthodontics and implant dentistry. It evaluates AI’s potential to enhance diagnostic accuracy, treatment planning, and prosthesis fabrication while addressing critical ethical considerations such as data security, algorithm bias, and access disparities. Additionally, this review contextualizes the significance of these advancements within the broader challenges of integrating AI into dental care, highlighting both its clinical potential and the ethical challenges it presents. By synthesizing current evidence on clinical applications, performance outcomes, ethical concerns, and proposed resolutions, this review underscores the importance of advancing responsibly while addressing ethical implications to ensure the responsible adoption of AI technologies in prosthodontics and implant dentistry.

## 2. Materials and Methods

The review used Preferred Reporting Items for Systematic Review and Meta-analysis Principles (PRISMA) guidelines. The search was conducted on PubMed, Medline, Web of Science, and Scopus databases. In addition, a manual search was performed. The articles in English published from January 2022 to 30 May 2024, were included.

The search strategy was developed using specific keywords and Boolean operators to combine terms effectively. The primary search terms included “Artificial Intelligence”, “Deep Learning”, “Machine Learning”, “Neural Networks”, “Prosthodontics”, “Dental Implants”, “Implantology”, and “Ethics”. These terms were combined using Boolean operators (AND/OR) to refine the results and target studies addressing the use of AI in prosthodontics and implant dentistry. For example, the following search string was used: (“Artificial Intelligence” OR “Deep Learning” OR “Machine Learning” OR “Neural Networks”) AND (“Prosthodontics” OR “Dental Implants” OR “Implantology”) AND (“Ethics” OR “Ethical Considerations”). The search strings were tailored to the indexing system of each database to ensure maximum coverage and accuracy. Synonyms and additional terms such as “Algorithm Bias” and “Data Privacy” were incorporated where necessary to capture a broader range of studies.

The literature search was performed on 30 May 2024. All titles and abstracts that evaluated the impact of AI-based tools on performance outcomes and ethical considerations were compared to traditional methods in prosthodontics and implant dentistry. A population or problem, intervention, comparison, and outcome (PICO) question was formulated. The research question was “How do AI-based tools compare to traditional methods in terms of performance outcomes and ethical considerations in prosthodontics and implant dentistry?” The population (P) included any patient or extracted teeth with clinical applications in prosthodontics and implant dentistry; intervention (I) included the use of AI-based tools; comparison (C) was made between AI-based tools and traditional methods; outcome (O) included AI-based tools performance outcomes and ethical considerations. The inclusion criteria were original research studies published in English that evaluated the application of AI-based tools in prosthodontics and implant dentistry, focusing on performance outcomes or ethical considerations. Literature reviews, letters to editors, non-English publications, and articles with inaccessible full-text papers were excluded from the review.

Two reviewers independently screened titles and abstracts of all studies retrieved from the search mentioned above strategy. The abstracts of the full-text articles were examined based on their relevance and eligibility for inclusion. Reasons for exclusion during full-text screening included (1) lack of focus on prosthodontics or implant dentistry, (2) absence of AI-specific analysis, and (3) inaccessible full-text articles.

Following this, full-text screening was performed by one of the independent reviewers and assessed by another reviewer. Disagreements between reviewers were resolved by discussion. In addition, a manual search was conducted to seek additional articles for inclusion. The quality assessment and risk of bias of included studies were evaluated with the Newcastle–Ottawa Scale [11].

## 3. Results

### 3.1. Applications of AI in Prosthodontics and Implant Dentistry

**Figure 1:**
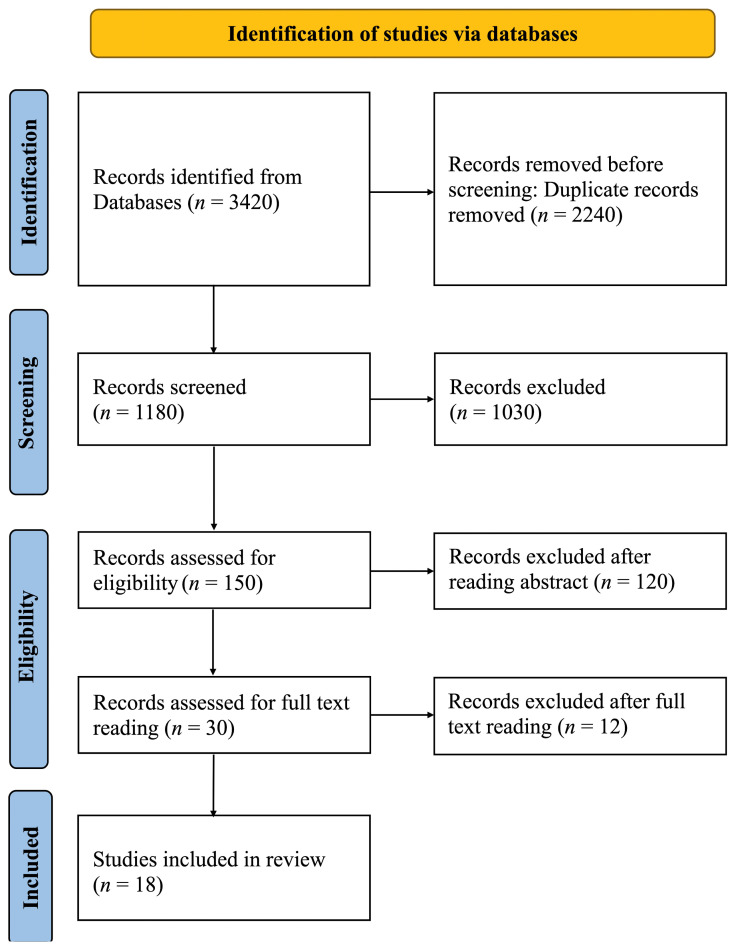
PRISMA flow diagram of studies discussing applications of AI in prosthodontics and implant dentistry.

The PRISMA flow diagram’s visually summarized article screening process is shown in Figure 1. Articles related to AI tool applications outside of prosthodontics and implant dentistry were excluded. A total of 3420 articles were identified after searching for electronic sources. After the removal of duplicates, 1180 articles were identified. After screening with titles, 1030 articles were considered irrelevant, and the remaining 150 studies were considered for abstract reading. Of these, 120 studies were rejected after reading the abstract, and the remaining 30 were considered for full-text reading. Lastly, 18 studies that met the eligibility criteria were included in the review. Due to the heterogeneity of the critical parameters, aggregation of statistical data was impossible. Therefore, a descriptive analysis of the studies that were obtained was conducted.

#### 3.1.1. Characteristics of the Included Studies

Table 1 summarizes the included studies related to applications of AI in prosthodontics and implant dentistry. Out of the 18 studies reviewed, 16 were retrospective. These studies, including those by Kong, H.J. et al., Alsomali et al., Kurtulus et al., Wang et al., Al-Asali et al., Fontenele et al., Seok Oh et al., Moufti, M.A. et al., Park, W. et al., Park J.H. et al., Kim et al., Sukegawa et al., Kong, H.J., Al-Sarem et al., Altan et al., and Sakai, T. et al., utilized existing data to evaluate AI techniques and models [12,13,14,15,16,17,18,19,20,21,22,23,24,25,26,27]. By analyzing pre-recorded data, these studies assessed the performance and application of AI in various dental tasks. In contrast, the study by Lyakhov, P.A. employed a descriptive approach, focusing on predicting implant survival rates rather than analyzing pre-existing data [28]. Meanwhile, Chau et al. conducted a feasibility study to explore AI’s application in designing dental prostheses [29] This study aimed to assess the accuracy of a new AI system for creating biomimetic single-molar dental prostheses and compare them to natural molar teeth.

Among the 18 studies, 8 used various imaging modalities to classify dental implants or prostheses [12,14,20,21,22,23,24,26]. Four studies focused on predicting implant success or treatment plans using AI models [13,18,27,28]. Additionally, five studies, by Wang et al., Al-Asali, M. et al., Fontenele et al., Moufti et al., and Al-Sarem et al., developed AI tools for bone or tooth segmentation [15,16,17,19,25]. Among these studies, Moufti, M.A. et al. developed an AI tool to identify and outline missing teeth areas on CBCT images before placing implants. Chau et al. focused on using AI to design single-molar dental prostheses and compared these designs to natural molar teeth [29].

**Figure 2:**
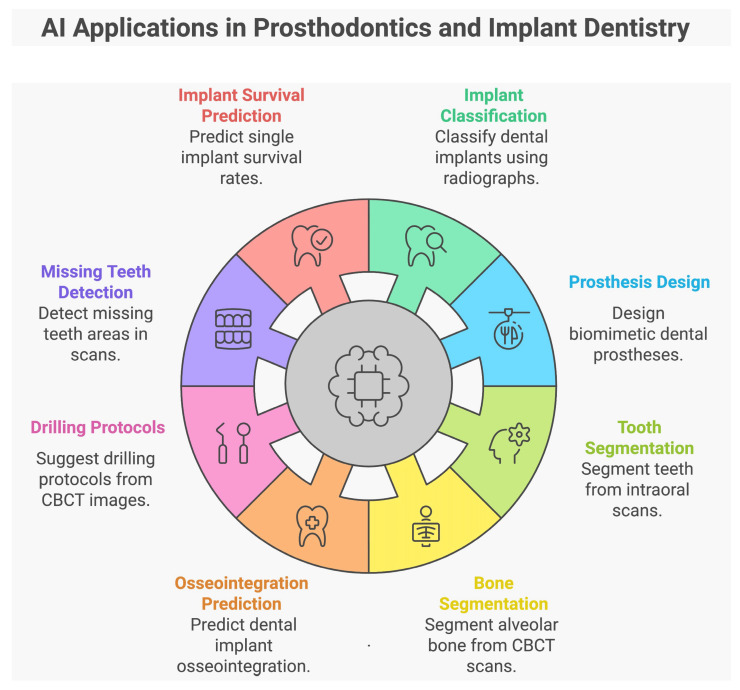
Applications of AI in prosthodontics and implant dentistry. This figure was generated using a free AI tool, Napkin AI (https://www.napkin.ai/, accessed on 6 October 2024).

Figure 2 summarizes the applications of AI in prosthodontics and implant dentistry.

Table 2 summarizes the quality assessment and risk of bias, as evaluated using the Newcastle–Ottawa Scale, in studies related to the applications of AI in prosthodontics and implant dentistry. Higher scores indicate a lower risk of bias. Most studies score between 5 and 6, indicating a moderate risk of bias. The study by Altan et al. stands out with a total score of eight, indicating a lower risk of bias, as this study likely addressed more of the quality and comparability criteria than the others [26]. The study by Oh et al. also scores slightly better (six), suggesting moderate attention to bias control, though issues such as follow-up remain [18]. In summary, the majority of the studies evaluated here show moderate risks of bias, primarily due to selection concerns and insufficient follow-up duration. This implies that while AI is being increasingly applied in prosthodontics and implant dentistry, there are limitations in the robustness and design of the studies, which could impact the reliability of their findings.

#### 3.1.2. Type of Dataset Utilized to Extract Data

Among the given studies, four studies by Kong, H.J. et al., Kurtulus et al., Sukegawaet et al., and Altan et al. utilized panoramic radiographs to extract and assess data [12,14,23,26]. Another six studies by Alsomali et al., Al-Asali M et al., Fontenele et al., Al-Sarem et al., Sakai et al., and Moufti, M.A. et al. utilized CBCT images for the analysis [13,16,17,25,27]. Meanwhile, three studies used periapical radiographs to extract data [21,22,24]. Whereas Seok et al. and Park et al. utilized both panoramic and periapical radiographs to extract and analyze data [18,20]. Moreover, Wang et al. and Lyakhov et al. utilized intraoral scanner images and patient histories, respectively, to extract data [15,28]. Meanwhile, Chau et al. utilized maxillary cast models to study and design the single molar prostheses for the study [29].

#### 3.1.3. Total Number of Datasets Extracted and Utilized for Training and Testing AI

In analyzing the dataset utilization across various dental imaging and AI studies, Kurtulus et al. used 1258 panoramic radiographs, allocating 80% to training and 10% to testing, which supports thorough model evaluation [14]. Kong, H.J. et al. utilized a substantial dataset of 14,037 panoramic radiographs, dedicating 80% for training and 20% for testing, ensuring robust model development and validation [12]. Sukegawa et al. analyzed 10,191 dental implant images, though the training/validation split was not specified [23]. Altan et al. used 5126 panoramic radiographs with a split of 90%/10% into training and testing sets [26].

Alsomali, D. et al. worked with 16,272 images from 34 CBCT cases, with 90.2% of images used for training and 9.8% for testing, highlighting an extensive training set [13]. Al-Sarem et al. utilized 500 CBCT images, divided into 70% for training, 20% for validation, and 10% for testing [25]. Fontenele, C.R. et al. focused on 141 CBCT scans, splitting them 70%/30% [17]. Moufti, M.A. et al. worked with 43 CBCT images, using 33 for training and 10 for validation [19]. Al-Asali, M. et al. utilized 150 CBCT images, with an 80%/20% split [16]. Lastly, Sakai, T. et al. utilized 1200 images with an 80%/20% split [27].

In contrast, Kim et al. worked with 263 periapical radiographs from 355 implant fixtures, following an 80%/20% split [22]. Another study by Kong, H.J. utilized 4800 periapical radiographs with an 80%/10%/10% split [24]. Park, J.H. et al. analyzed 1320 images from periapical radiographs, using 960 images for training and 180 each for validation and testing [21].

Meanwhile, Park, W. et al. managed a large-scale dataset of 156,965 images, which included 116,756 panoramic and 40,209 periapical images, with 80% used for training and 10% each for validation and testing, demonstrating a comprehensive approach to model development [20]. In addition, Seok Oh et al. studied 1206 implants from panoramic and periapical radiographs, dividing the data into 60% for training, 20% for validation, and 20% for testing [18].

Wang et al. employed 761 IOS images, with 609 for training and 76 for validation [15]. Unlike other studies that used digital imaging for data assessment, Chau, R.C.W. et al. studied data from 169 participants’ maxillary casts, with 159 casts used for training, and the remaining 10 for validation [29]. Lyakhov, P.A. et al. focused on 1646 patient histories, with an 80/20 split between training and testing, though specific image counts are not detailed [28].

#### 3.1.4. Object Validation Method Utilized

The 18 studies on AI applications in dental radiography and imaging used object validation methods to ensure accurate and effective analysis. Kong, H.J. et al. utilized YOLO versions 5 and 7 for object detection in panoramic radiographs, highlighting YOLO’s strength in real-time object detection tasks [12]. Altan et al. used the YOLOv4 model for object detection in panoramic radiographs of crowns and bridges, emphasizing YOLOv4′s accuracy and speed [26]. Meanwhile, Kim et al. used YOLOv3 to detect and locate implant fixtures in periapical radiographs [22].

Kurtulus et al. utilized a variety of CNN models, including VGG16, ResNet-50, EfficientNet, Vovnet 57, Vovnet 39, and ConvNeXt, providing a comprehensive approach to feature extraction and classification in panoramic radiographs [14]. Al-Sarem et al. employed a range of CNN models, including AlexNet, VGG16, VGG19, ResNet50, DenseNet169, and MobileNetV3, to handle CBCT images, showcasing the versatility of these architectures [25]. Alsomali, D. et al. implemented Mask R-CNN for CBCT cases, particularly effective for object instance segmentation [13]. Lyakhov, P.A. et al. utilized the PyTorch machine learning framework to validate their CNN-based model on patient histories, ensuring flexibility and robustness in model implementation [28]. Sukegawa et al. employed Attention-Based Networks (ABN) and ResNet variants (ResNet18, ResNet50, ResNet152) to analyze dental implant images [23]. Park, J.H. et al. utilized VGG16 and the k-means++ algorithm for deep learning and clustering analysis, integrating deep learning with unsupervised learning methods for better classification [21]. Seok Oh et al. employed ResNet and DenseNet variants to analyze dental implant panoramic and periapical radiographs, leveraging these networks’ deep feature extraction capabilities [18]. Finally, Sakai, T. et al. applied the LeNet-5 model, a CNN architecture, for validating dental images [27].

Park, W. et al. used an automated DL algorithm to validate a large dataset of panoramic and periapical images, ensuring scalability and efficiency [20]. Another study by Kong, H.J. utilized AutoML, an automated machine learning tool, for validating periapical radiographs, enabling efficient model selection and optimization [24].

Moufti, M.A. et al. used U-Net, a popular architecture for biomedical image segmentation, to validate CBCT images [19]. Similarly, Al-Asali, M. et al. used two versions of the U-Net model for segmenting CBCT images, demonstrating its effectiveness in detailed segmentation tasks [16]. Meanwhile, Wang et al. employed a combination of 3D U-Net and CNN models for validating IOS images, enhancing 3D image processing capabilities [15]. Fontenele, C.R. et al. utilized the Virtual Patient Creator, a novel method for generating and validating CBCT data [17]. Lastly, Chau, R.C.W. et al. applied a 3D Generative Adversarial Network (GAN) for validation, demonstrating the use of generative models in medical image analysis [29].

#### 3.1.5. Experts Involved in Data Validation

The validation of data in the 18 studies demonstrates a variety of approaches. Several studies such as Alsomali, D. et al., Kim et al., Sukegawa et al., Al-Asali, M. et al., Altan et al., Wang et al., Kong, H.J., and Sakai, T. et al. did not specify their data validation methods and are marked as “Not Reported Data” (NRD) [13,15,16,22,23,24,26,27]. Conversely, some studies involved specific experts for validation. For example, Kong et al. and Park, J. H. et al. employed experienced prosthodontists [12,21]. Another study by Park, W. et al. utilized oral and maxillofacial radiologists and implantologists for data validation [20]. Fontenele, C.R. et al. [17]. used two operators, while Moufti, M.A. et al. involved two students and one dental specialist [19]. Chau, R.C.W. et al. relied on blinded assessors [29], and Lyakhov, P.A. et al. and Kurtulus et al. engaged physicians and specialists in dental implantology [14,28]. Seok Oh et al. and AL-Sarem worked with a dental surgeon [18,25]. This variety in validation approaches reflects the different methodologies and the emphasis on ensuring accuracy and reliability in the studies.

#### 3.1.6. Performance Metrics Evaluation

The parameters evaluated across these 18 studies encompass a broad spectrum of metrics to assess various performance aspects. Accuracy, precision, and recall are frequently evaluated metrics, often alongside the F1-score, as seen in studies by Kurtulus et al., Park, W.et al., Al-Sarem et al., Sukegawa et al., and Kong, H.J. [14,20,23,24,25]. Meanwhile, Alsomali et al. utilized several sections and correct/incorrect identification of GP markers [13], and Lyakhov et al. evaluated 55 patient factors, respectively, to evaluate the AI performance [28]. Altan et al. utilized recall, mean average precision (mAP), mean average recall (mAR), and F1 score for performance analysis [26]. Kong, H.J. et al. employed mAP to gauge performance [12]. Advanced metrics like Hausdorff distance (HD), intersection over union (IoU), and dice similarity coefficient (DSC) are used to measure spatial accuracy and overlap, as seen in Fontenele, C.R. et al. and Wang et al [15,17]. At the same time, sensitivity, specificity, and area under the receiver operating characteristic (ROC) curve are evaluated by Seok Oh et al. and Park, J. H. et al. to assess diagnostic performance [18,21]. Kim et al. utilized sensitivity, specificity, accuracy, and confidence score [22]. Other metrics such as the average HD, volume similarity (VS), mean surface distance (MSD), and standard deviation (SD) were included in a study by Al-Asali, M. et al [16]. Studies like Chau, R.C.W. et al. utilized the average HD between each AI-designed tooth and the original tooth [29]. Moufti et al. utilized the DSC to evaluate the performance metrics [19]. Including these diverse parameters highlights the comprehensive approach taken to assess performance across different methods and contexts.

### 3.2. Ethical Issues Associated with the Use of AI in Dentistry

**Figure 3:**
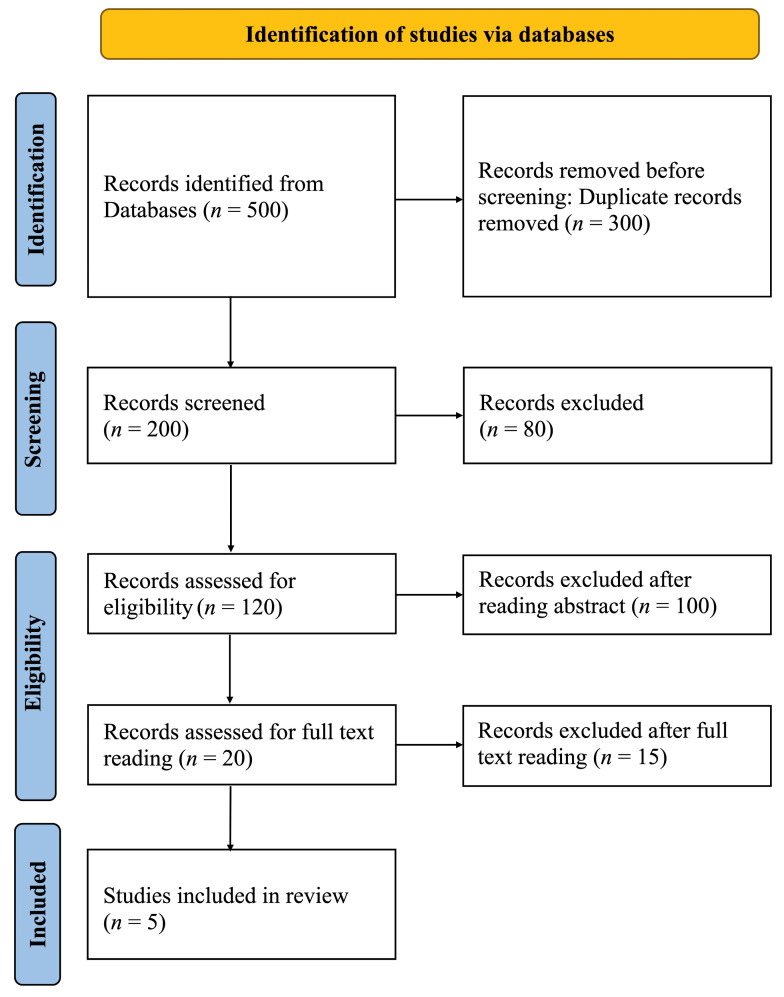
PRISMA flow diagram of studies discussing ethical considerations related to AI in dentistry.

The PRISMA flow diagram’s visually summarized article screening process is shown in Figure 3. To include more studies related to ethical considerations, all articles related to dentistry were included. A total of 500 articles were identified through searches of electronic sources. After removing duplicates, 200 articles remained. Following initial screening, 80 articles were deemed irrelevant, and the remaining 120 studies were considered for abstract review. Of these, 100 studies were excluded after reading the abstracts, and the remaining 20 were selected for full-text review. Ultimately, fifteen articles were excluded for various reasons, and five studies were included in the review. Due to the heterogeneity of the key parameters, aggregating statistical data was not possible; therefore, a descriptive analysis of the included studies was conducted.

#### Characteristics of Included Studies

There are relatively few studies that address the ethical issues associated with the use of AI in dentistry. The Table 3 summaries of included studies relate to ethical considerations of AI applications in dentistry. Existing literature mainly focuses on the perspectives of patients or dental professionals, often covering only patient consent and privacy concerns. Kosan, E. et al. (2022) conducted an observational study analyzing patients’ views on AI in dentistry. Out of 165 approached participants, 17 refused to participate, 3 did not return the questionnaire, and 5 submitted incomplete surveys, resulting in a final sample size of 140. Participants aged 18 to 84 included 69 males (49.3%) and 71 females (50.7%). The study used the dentalXrai AI system, with patients shown conventional and AI-enhanced radiographs that highlighted carious lesions in different colors for easier identification. Results indicated that participants found AI useful (4.19 ± 0.8), were not generally afraid of AI (2.23 ± 1.0), and specifically felt no fear of AI in dentistry (1.65 ± 0.8). The study concluded that AI-based communication significantly improved patients’ ability to detect caries in radiographs [30].

A recent cross-sectional study by Hamd, Z.Y. et al. (2023) examined the attitudes, knowledge, and willingness of dental professionals and students regarding AI in dentistry. The survey, divided into two sections—one for demographic data and one for knowledge, perception, and readiness to integrate AI into practice—received 134 valid responses. Of these, 64.9% were female, 53.7% were undergraduate students without clinical experience, 14.2% were academicians, and 32.8% were clinical dentists. Among participants, 73.1% had completed their highest degree in UAE universities, while 26.9% studied abroad. The study found that 85.5% believed AI would play a significant role in practice, 85.1% thought AI would be used in many dental applications, and 82.1% were ready to learn and apply AI. However, only 42.5% understood AI, 29.1% were familiar but hesitant to use it, and 9.7% were unfamiliar with its workings. Overall, 85.5% of dental professionals acknowledged AI’s importance, while 31.3% felt it might threaten or disrupt their profession [31].

Similarly, Ayad, N. et al. (2023) conducted an observational survey exploring patients’ perspectives on AI in dentistry. The survey included 265 patients who provided data on demographics and self-perceived dental health. While 93.6% rated their knowledge of digital technology as average or above, only 52.5% rated their knowledge of AI similarly, and 47.5% rated it below average. Concerns about AI included its potential impact on workforce needs (37.7%), trust between dentists and patients (36.2%), and treatment costs (31.7%). On the positive side, 60.8% believed AI would enhance diagnostic confidence, 48.3% thought it would reduce time, and 43% felt it would lead to more personalized and evidence-based disease management. Overall, 73.6% of participants agreed that AI would benefit dentistry [32].

Roganovic, J. et al. (2023) conducted a cross-sectional online survey involving experienced dentists and final-year dental students. Of 281 invitations, 193 responses were received (58.5% students and 77.5% experienced dentists). The survey found that 27.1% of participants were unfamiliar with AI in dentistry, and only 10.9% used AI in practice. Concerns about being replaced by AI were expressed by 8% of participants (yes) and 35% (partly). The study concluded that dentists and students were skeptical about AI, with students particularly anxious about potential replacement and the lack of regulatory policies [33].

Rokhshad, R. et al. conducted a study in 2023 to identify the ethical challenges of using AI in smile designing. The ethical principles considered in the study were wellness, respect for autonomy, privacy protection, solidarity, governance, equity, diversity, expertise/prudence, accountability/responsibility, sustainability, and transparency. The participants included in the study group were dentists who had a background of researchers, journal editors, reviewers, methodologists, policymakers, and educators in the field of AI in dentistry, and all of them were from different countries. In the first round using e-Delphi process, out of the 11 ethical challenges, the participants agreed on 7 of them (diversity, transparency, wellness, privacy protection, prudence, law and governance, and sustainable development) and disagreed on 4 principles (equity, accountability and responsibility, solidarity, and respect for autonomy); therefore, further discussions were carried out for its reformulation. After reformulating the statements regarding the four principles that were previously in disagreement, the participants reached an agreement in round two. This study concluded that AI software for smile designing should adhere to these ethical principles to ensure better acceptance [34].

**Figure 4:**
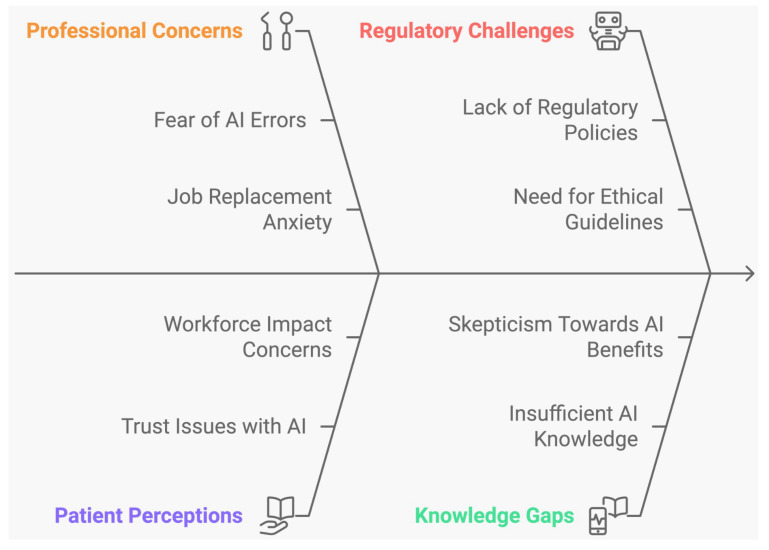
Ethical issues associated with the use of AI in dentistry. This figure was generated using a free AI tool, Napkin AI (https://www.napkin.ai/, accessed on 6 October 2024).

Figure 4 shows a summary of the ethical issues associated with the use of AI in dentistry.

Table 4 summarizes the quality assessment and risk of bias, as evaluated using the Newcastle–Ottawa Scale, in studies related to ethical considerations of AI applications in dentistry. Higher scores indicate a lower risk of bias. Most studies score four, indicating a moderate-to-high risk of bias across the evaluated trials. Kosan, E. et al. have a slightly higher total score of five, indicating a relatively lower risk of bias compared to the others, but still with significant limitations [30]. Overall, this table highlights significant ethical concerns with the studies, primarily revolving around poor follow-up, lack of control for confounders, and low generalizability of results. The total scores suggest that the studies, while relevant, exhibit considerable risk of bias, which could limit their impact or the conclusions that can be drawn from them regarding AI ethics in dentistry.

## 4. Discussion

This review offers a comprehensive analysis of current artificial intelligence applications in prosthodontics and implant dentistry, addressing both their advancements and the ethical challenges they pose. It highlights key findings from cross-sectional studies on these ethical issues and introduces an ethical framework aimed at enhancing the reliability of AI in dentistry. Given the rapid evolution of this field, the period from January 2022 to May 2024 was selected to include the most up-to-date, valid, and impactful studies, as older research may lack relevance due to outdated AI technologies or methodologies.

### 4.1. Applications of AI in Prosthodontics and Implant Dentistry

Among 18 studies that evaluated the treatment outcomes of using AI in the field of prosthodontics and implantology, 8 studies evaluated the performance of AI in dental implant classification. Among these studies, Kong, H.J. et al. demonstrated that deep learning models for implant design classification are highly effective, with performance being influenced by algorithm type, image processing, and design details [12]. Kurtulus et al. showed that CNN models can accurately classify dental implant systems from panoramic radiographs. Park et al. noted that automated deep learning provides reliable classification with large datasets, although no significant accuracy difference was found between panoramic and periapical images [14]. Another study by Park, J. H. et al. observed reliable classification performance with deep learning and clustering analysis AI models [21]. Altan et al. reported that deep learning methods detected prosthetic restorations with high accuracy [26]. Moreover, Kim et al. achieved high performance in classifying implant fixtures with the deep learning YOLOv3 model through transfer learning, even while working with a limited dataset [22]. Kong, H.J. demonstrated that AutoML AI models on cloud platforms are effective for high-accuracy dental implant classification [24]. Meanwhile, Sukegawa et al. reported excellent performance and high compatibility of the convolutional neural network ResNet18 with the ABN model in dental implant classification [23]. These results imply that the use of deep learning models, including various algorithm types and neural network architectures, has displayed an illustrious performance in the classification of dental implants. Although both automated and cloud-based AutoML models have demonstrated high accuracy, the selection of the model and data type remains important.

In addition, four studies by Seok-OH et al., Alsomali et al., Lyakhov, and Sakai et al. used AI models to predict implant success or plan treatment [13,18,27,28], Alsomali, D. et al. reported that training AI with only axial images is insufficient for accurate performance [13]. Seok Oh et al. found that deep learning can partially predict dental implant osseointegration from plain radiography [18]. Sakai, T. et al. highlighted that AI models effectively predict drilling protocols from CBCT images, supporting pre-surgery decision-making for achieving primary stability [27]. Meanwhile, Lyakhov, P.A. et al. observed that neural network systems for analyzing patient factors provide higher accuracy for single implant predictions but lack comprehensive decision support capabilities [28]. These findings suggest that AI models trained on limited data, such as only axial images or plain radiography, rather than a variety of images (such as panoramic, periapical, CBCT, and intraoral images) or patient factors based on case histories, tend to perform less accurately and negatively impact clinical decision-making, indicating the need for training on more diverse datasets.

Another five studies by Fontenele et al., Al-Sarem et al., Al-Asali, M. et al., Wang et al., and Moufti, M.A. et al. developed AI tools for bone or tooth segmentation [15,16,17,19,25]. Wang et al. demonstrated that the 3D U-Net pipeline provided accurate and efficient automated tooth segmentation on IOS images [15]. Al-Asali, M. et al. found U-Net models effective for segmenting bone in regions with missing teeth in cone-beam computed tomography (CBCT) scans and for predicting implant positions, thereby aiding in automated implant planning [16]. Fontenele, C.R. et al. reported that manual segmentation was slightly more accurate than CNN-based tools, though the CNN tool was 116 times faster [17]. Moufti, M.A. et al. noted that their model accurately segmented edentulous bone areas, potentially reducing time and costs in implant treatment [19]. Al-Sarem et al. identified DenseNet169 as a promising, time-efficient tool for automated dental implant planning [25]. These findings suggest that DenseNet169 and U-Net models are effective for automated planning and segmentation. Although manual segmentation might offer slightly better accuracy, CNN-based tools are much faster, highlighting the compromise between speed and precision. Finally, Chau, R.C.W. et al. successfully designed a dental prosthesis using a 3D GAN AI system that mimicked natural tooth morphology, achieving high performance even with limited data [29].

Overall, the inference derived from these studies indicates that the application of AI has substantially advanced the field of dental implant and prosthodontics in the diagnosis and planning stages. The AI technologies used in prosthodontics and implant dentistry are summarized in Table 5.

**Figure 5:**
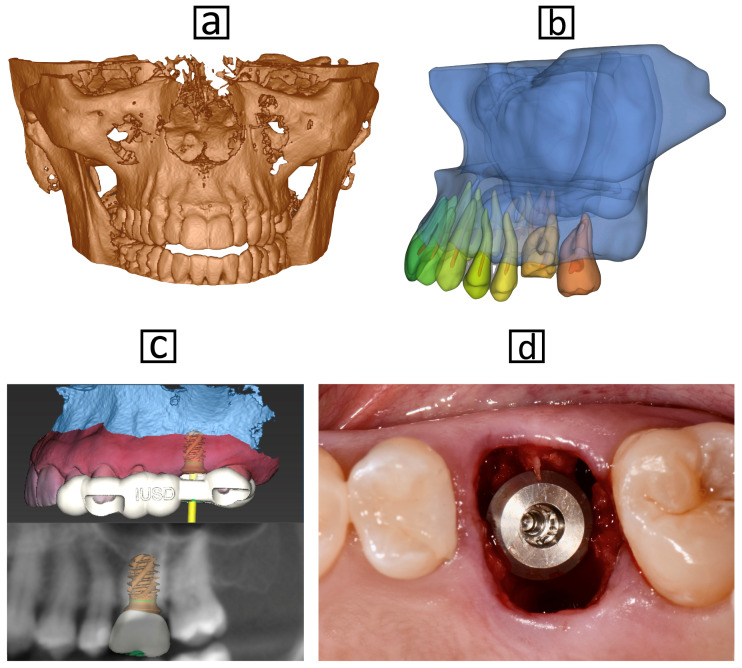
(**a**) Before segmentation, it is not possible to distinguish anatomical structures in the CBCT images. (**b**) AI-based automatic segmentation isolating and defining specific anatomical structures within CBCT images efficiently. The segmented dataset can be used for diagnosis, treatment planning, or implant site evaluation, such as extraction and immediate implant placement for the maxillary left first molar in this example. (**c**) Virtual implant planning using CBCT scans, segmentations, and intraoral scans. (**d**) Immediate implant placement.

AI has proven effective in providing reliable classifications, CBCT segmentation for implant planning and site evaluation, predicting implant success, and suggesting drilling protocols for implant placement. The clinical examples of AI automatic CBCT segmentation are shown in Figure 5.

This advancement has streamlined presurgical procedures, enhanced accuracy, and efficiency, and offered valuable support to clinicians in clinical decision-making. Consequently, this has contributed to reducing both the cost and time involved in performing dental implant procedures. Nevertheless, further refinement and the incorporation of diverse data types are essential for improving performance across various clinical scenarios.

#### Limitations of Studies

Kong, H.J. et al. highlighted that while the accuracy of object detection models was high, actual implant identification was not evaluated, particularly for implant systems with similar design features where detection could be challenging. The images used for model learning were collected from various dental clinics without standardization, which could degrade model performance [12]. Another study by Kong, H.J. noted that the study’s limited implant types, particularly internal and external implants, may have negatively affected confidence in the study’s accuracy [24]. Kurtulus et al. observed that an unequal number of datasets for each brand represented a significant limitation [14]. Park, W. et al. and J.H. Park emphasized the need for larger and more diverse datasets from multiple centers to improve model generalizability and clinical applicability [20,21]. Kim et al. noted that their study used approximately 1900 images, which was insufficient for recognizing all types of prostheses used in dental procedures [22]. Swegawa et al. highlighted that the CNN models considered required substantial computational resources, and there was a need for further validation to determine compatibility with specific models [23]. Al-asali et al. discussed limitations related to manual cropping and 3D Slicers for missing tooth segmentation [16]. Lyakhov noted that the proposed neural network system could only serve as an adjunct diagnostic tool rather than a complete decision-making aid [28]. Seok Oh et al. and Alsomali et al. identified issues with axial images lacking clear and distinct marker shapes, which could hinder AI model performance in predicting osseointegration and identification of GP markers, respectively [13,18]. Fontenel et al. pointed out that CNNs were trained and validated with images from only two CBCT devices, limiting generalizability to other devices, and that the anatomical focus was restricted to the maxillary region to minimize manual labeling errors [17]. According to Chau et al., even though the AI system successfully designed a single-tooth dental prosthesis that mimicked a natural tooth [29], it was created in a computer-simulated environment that did not account for clinical factors such as drifting, tilting, or supra-eruption of adjacent teeth. These collective limitations highlight the need for more standardized, diverse datasets, and refined methodologies to enhance the accuracy and clinical applicability of AI models in the field of prosthodontics and implant dentistry.

The limitations across various studies on implant identification using deep learning methodologies are multifaceted. Many studies in this review are retrospective in nature, which inherently limits the ability to establish causality and generalizability in real-world clinical settings. This is particularly significant as the topic of artificial intelligence in prosthodontics and implant dentistry is relatively recent, resulting in a scarcity of high-quality prospective trials. The lack of longitudinal and diverse datasets across studies also impedes the ability to validate AI tools comprehensively in varied clinical environments. Another significant limitation hindering the widespread adoption of AI in dentistry is the lack of interoperability among AI systems [35]. This issue arises due to the absence of standardized protocols for data exchange, model integration, and compatibility across different AI platforms [35]. For example, AI systems developed by various vendors may use proprietary algorithms or data formats that are not compatible with each other, making it difficult to integrate these systems into existing clinical workflows [36]. Addressing this challenge requires establishing industry-wide standards and frameworks, similar to those used in other medical domains, to ensure seamless integration and communication between AI systems. Overcoming this limitation is crucial for facilitating broader adoption and maximizing the clinical utility of AI in dentistry [35,36].

### 4.2. Ethical Issues Associated with the Use of AI in Dentistry

In this study, only a small number of the included research addressed potential ethical concerns. Of the five studies that evaluated the ethical considerations of AI in dentistry, Kosan et al. found that patients generally had a positive attitude toward AI, and that AI-based diagnostics did not impact their trust in the technology [30]. The study indicated that AI-based instruments enhanced communication of radiographic findings and helped patients identify carious lesions on radiographs. Similarly, Ayad et al. reported a positive patient attitude toward AI, noting that its use in dentistry can increase diagnostic confidence, improve time efficiency, and provide more personalized disease management [32]. In contrast, Hamd, Z.Y. et al. reported that while the majority of healthcare professionals view AI as a promising development in the field, others consider it unreliable and incapable of replacing human healthcare providers. Additionally, a small segment of healthcare professionals sees AI as a threat to their profession [31]. This underscores the need for dental professional societies and educational institutions to collaborate in developing comprehensive training programs to address these concerns and bridge the knowledge gap. Similarly, Roganovic et al. reported that only a small segment of experienced dentists and final-year dental students were familiar with AI and supported its use in dentistry [33]. This lack of support was attributed to insufficient knowledge, fear of replacement, and inadequate regulatory policies. Additionally, female dentists expressed more ethical concerns regarding AI compared to their male counterparts. The study underscores the need for comprehensive training and the inclusion of regulatory policies to facilitate the adoption of AI in dental practice. Lastly, Rokhshad et al. simulated and identified potential ethical challenges associated with the use of AI software for smile design by clinicians and patients [34]. This research is the first to address these ethical issues related to AI in dental treatment procedures. It fosters discussions aimed at enhancing the reliability of AI applications in dentistry.

Overall, these cross-sectional surveys reveal that both patients and dental professionals have limited knowledge about AI in dental practice and express concerns regarding data privacy, system bias, regulatory gaps, and the potential for AI to replace human practitioners. Addressing these issues through improved knowledge and training programs for students and professionals could mitigate these concerns and facilitate better integration of AI into clinical settings.

#### Limitations of Studies

The study by Kosan et al. relied solely on hypothetical scenarios, for instance, asking participants to imagine having dental caries, which raises concerns about the real-life applicability of the findings and the inability of patients to express actual experience [30]. Moreover, as the study was conducted during the COVID-19 peak in Germany, the study’s sample was limited and skewed toward healthier individuals, impacting the generalizability of the study. Hamd et al. emphasized the shortage of literature on this topic as a limitation, suggesting that incorporating open and focus group discussions could enhance a more comprehensive understanding [31]. Ayad et al. pointed out that since the study was performed in Germany, a high-income country with high oral care awareness, it might not be representative of other settings or developing or underdeveloped nations [32]. Roganovic et al. found that the study’s geographical limitation and the lack of sample size estimation and sensitivity analyses were additional constraints [33]. Rokhshad et al. highlighted that the study’s e-Delphi participants included only a small subset of routine users of smile design software, potentially limiting the generality of the results. The study’s European context, influenced by the EU AI Act, might affect the generalizability of the ethical considerations to other countries with different data protection and AI ethics frameworks [34]. These studies have only been validated internally, which may limit the generalizability of their findings. Therefore, further research with larger sample sizes is needed to validate the results.

### 4.3. Ethical Framework in Dentistry

The implementation of AI in dentistry necessitates rigorous ethical oversight to prevent harm and address the moral considerations associated with these technologies [37]. Fiske et al. reported that the adoption of AI could render traditional dental services obsolete, potentially increasing disparities in oral health care [38]. Additionally, the large-scale data sharing required for AI integration poses significant risks to patient privacy [39]. Data used to calibrate algorithms could eventually be commercialized, potentially leading to additional costs for patients when they access the technology [40]. Furthermore, in cases where AI systems generate errors in clinical settings, these systems cannot be held accountable, whether they are used independently or under supervision [41]. The rise of unsupervised, chatbot-based diagnostic tools makes it increasingly challenging to apply human ethical standards to AI [42]. Therefore, there is an urgent need for a comprehensive ethical framework to govern the use of AI in clinical settings.

Before addressing the ethical considerations specific to AI, it is essential to understand the foundational ethical principles that guide healthcare practice. These principles include autonomy, non-maleficence, beneficence, and justice. Autonomy ensures that patients receive comprehensive information about their treatment options. With this information, patients should be able to make informed decisions without undue influence from clinicians [43,44]. Non-maleficence requires clinicians to avoid causing harm to patients. In contrast, beneficence focuses on promoting the well-being of patients. Finally, justice involves the fair distribution of resources, ensuring affordability, and prioritizing care based on patients’ needs [43,44]. Besides the above-mentioned ethical principles, ethical considerations related to the use of AI in dentistry include the following: privacy and data security, bias and fairness, transparency, and clinical validation and regulation [43,44].

#### 4.3.1. Privacy and Data Security

Since AI algorithms rely on extensive patient data for training, there are significant concerns regarding patient privacy and the protection of sensitive medical information. These concerns can be addressed by implementing stringent data security measures during data acquisition, storage, and algorithm development. Measures such as encryption, access controls, and strong authentication are crucial to prevent unauthorized access. Additionally, data minimization and anonymization should be implemented to regulate the handling of sensitive information. Compliance with privacy regulations, such as the Health Insurance Portability and Accountability Act (HIPAA) in the U.S. and GDPR (General Data Protection Regulation (GDPR) in the European Union) in the EU, is also critical [45]. These laws establish strict guidelines for managing protected health information and safeguarding patient rights. Furthermore, healthcare organizations must ensure transparency and accountability regarding data use. They should clearly communicate to patients how their data will be used and protected, maintaining adherence to ethical and legal standards [46].

#### 4.3.2. Bias and Fairness

Algorithmic bias can arise from the underrepresentation of diverse patient groups in training data, leading to discrepancies in diagnosis, treatment planning, and outcomes across diverse patient groups. This can exacerbate existing health inequalities and challenge the credibility and fairness of the healthcare system. To address these issues, it is crucial to enhance data diversity and inclusivity. For example, training AI algorithms with a broad range of patient demographics and using data augmentation techniques can help balance underrepresented groups [47]. Additionally, developers should employ fairness-aware techniques and transparent methods to detect and mitigate biases. Regular audits and evaluations by multidisciplinary teams are essential for monitoring and addressing biases, thereby restoring trust with patients and ensuring equitable AI-driven healthcare [48].

#### 4.3.3. Transparency

Transparency involves openly and clearly sharing information about how AI algorithms function and make decisions. The maintenance of transparency enables healthcare providers and patients to make informed decisions about patient care. Addressing this concern requires improving the interpretability of AI models and providing clear explanations for their outputs [43]. Various techniques including partial dependence plots, local interpretable model-agnostic explanations (LIME), and feature importance analysis can help elucidate the factors influencing algorithmic predictions [49]. Additionally, healthcare organizations should produce reports detailing the development and performance of AI models. Translating AI outputs into easily understandable language also aids patients in making informed decisions [43]. These measures collectively enhance trust, accountability, and acceptance of AI technologies.

#### 4.3.4. Clinical Validation and Regulation

AI algorithms present unique challenges for validation and regulation due to their reiterative development and their evolving development in healthcare data. Clinical validation provides a crucial role provide accurate, reliable, and clinically relevant results [43]. These challenges can be addressed by including robust testing protocols, clear guidelines for data collection, standardized performance metrics, and model development. Additionally, a combined effort among healthcare providers, technology developers, regulatory agencies, and patient groups is vital for developing consensus standards and best practices [50,51].

To mitigate the ethical challenges associated with AI, institutions can adopt regulatory frameworks and guidelines modeled after successful examples from other medical domains. For instance, the GDPR in the EU provides robust protections for patient data privacy, ensuring transparency and accountability in AI applications [52]. Similarly, the Food and Drug Administration (FDA) framework for AI-based medical devices in the U.S. emphasizes clinical validation, iterative testing, and risk management [53]. Institutions could adapt these principles by implementing standardized protocols for data handling, promoting inclusivity in AI training datasets, and fostering multidisciplinary collaboration to establish ethical oversight committees. These actionable solutions not only address algorithmic bias and data privacy but also ensure equitable and trustworthy AI integration into clinical practice [52,53].

## 5. Conclusions

The application of AI has significantly advanced the field of dental implantology and prosthodontics, enhancing diagnosis and treatment planning. AI has demonstrated its effectiveness in providing accurate classifications, predicting implant success, recommending drilling protocols, and assisting with basic prosthesis design. However, this rapid development also presents ethical concerns. Both patients and dental professionals often lack sufficient knowledge about AI’s role in dental practice and voice concerns about data privacy, system bias, regulatory shortcomings, and the fear that AI might replace human practitioners. These challenges highlight the risk of a “race to the bottom”, where technological progress outpaces ethical considerations. This review highlights AI’s transformative potential in prosthodontics and implant dentistry, emphasizing its ability to enhance clinical efficiency, accuracy, and outcomes. Despite these advancements, the ethical challenges associated with AI integration must be addressed through education and training programs, dental professionals can develop the knowledge and skills required to harness AI responsibly. Additionally, establishing regulatory frameworks to address data privacy, system fairness, and equitable access is essential to foster trust and widespread adoption. Furthermore, while AI has seen wider applications in implant diagnosis and planning, few studies have focused on its role in prosthodontics. Additional research is necessary to explore the full potential of AI in this area, ensuring that advancements in technology align with ethical considerations and clinical needs. The field must continue its “race to the moon” while staying grounded in ethical responsibility, balancing innovation with the fundamental principles of patient-centered care.

## Figures and Tables

**Table 1:** Summaries of included studies related to applications of AI in prosthodontics and implant dentistry.

Author, Year, and Country	Type of Study	Purpose of the Study	Total Number and Type of Dataset Utilized to Extract Data	AI Method	Method Used for Validation (Object Detection Algorithms)	Conclusion
Kurtulus et al. (2024) [14] Turkey	Retrospective	To evaluate the performance of deep learning techniques for classifying dental implant systems using panoramic radiographs.	1258 panoramic radiographs from 6586 implant systems.	Convolutional neural network (CNN) models	VGG16, ResNet-50, EfficientNet, Vovnet 57, Vovnet 39, and ConvNeXt.	All the proposed CNN models demonstrated capability to accurately classify dental implant systems from panoramic radiographs.
Chau, R.C.W. et al. (2024) [29] Hong Kong	Feasibility	To evaluate the accuracy of a new AI system in designing biomimetic single-molar dental prostheses and compare them to natural molar teeth.	169 participants	Convolutional neural network	3D Generative Adversarial Network (GAN)	3D GAN AI system successfully designed a single molar dental prosthesis that replicated the morphology of a naturally healthy tooth by learning the characteristics of the remaining dentition.
Wang et al. (2024) [15] Belgium	Retrospective	To evaluate the performance of a convolutional neural network (CNN) model for automatic tooth segmentation on intraoral scanner (IOS) images.	761 IOS images (380 upper jaws, 381 lower jaws) by Trios 3Shape intraoral scanner	Convolutional neural network	3D U-Net and CNN model	3D U-Net pipeline outperformed current methods for automated tooth segmentation on IOS images by providing accurate, efficient, and consistent results.
Al-Asali M. et al. (2024) [16] Saudi Arabia	Retrospective	To use U-Net models to segment missing bone areas in CBCT scans and predict implant positions.	150 CBCT images	Convolutional neural networks	U-net model 1 and 2	These models offer promising automated dental implant planning for dental implantologists.
Kong, H.J. et al. (2023) Republic of Korea [12]	Retrospective	To assess deep learning models’ performance in classifying and identifying 103 dental implant designs using panoramic radiographs.	14,037 images from Panoramic radiographs	Deep learning model	Versions 5 and 7 of You Only Look Once (YOLO) algorithm	A deep learning model for implant design achieved high performance, with mAP influenced by algorithm type, image processing, and design details.
Seok Oh et al. (2023) [18] Republic of Korea	Retrospective	To evaluate if deep learning can predict dental implant osseointegration using plain radiography.	Panoramic and periapical radiographs of 580 patients with 1206 implants	Deep learning model	ResNet-18,34,50, DenseNet-121,201 MobileNet-V2, and MobileNet-V3	Deep learning can help predict dental implant osseointegration using plain radiography to some extent
Park et al. (2023) [20] Republic of Korea	Retrospective	To assess the accuracy of automated deep learning model for identifying and classifying various types of dental implant systems (DIS).	Panoramic images: 116,756 Periapical images: 40,209 Total combined images: 156,965	Deep learning model	Automated DL algorithm	Automated deep learning demonstrated reliable classification accuracy with large datasets. However, no significant difference in accuracy between panoramic and periapical images was evident
Fontenele, C.R. et al. (2023) [17] Belgium	Retrospective	To develop and evaluate the efficacy of a new AI-driven CNN tool for automatically segmenting 3D maxillary alveolar bone from CBCT images.	141 CBCT Scans	Convolutional neural network	Virtual Patient Creator	Manual segmentation was slightly better than the CNN-based tool. However, newly introduced tool had higher accuracy and was 116 times faster than the manual tool.
Kong, H.J. et al. (2023) Republic of Korea [24]	Retrospective	To assess the accuracy and clinical usability of implant system classification using automated machine learning on the Google Cloud platform.	4800 periapical radiographs of 4 implant systems (Osstem TSIII, Osstem USII, Biomet 3i Osseotite External, and Dentsply Sirona Xive)	Deep machine	AutoML	The study demonstrated that AutoML AI model on a cloud platform is useful for the classification of dental implant systems with high accuracy.
Moufti, M.A. et al. (2023) [19] UAE	Retrospective	To develop and assess the efficacy of an AI tool to identify and outline areas of missing teeth on CBCT images before placing implants.	43 CBCT images	Convolutional neural network	U-Net	The model demonstrated high accuracy in segmenting edentulous bone areas compared to human investigators, potentially reducing the time and cost of implant treatment through automated CBCT image analysis.
Park, J.H. et al. (2023) [21] Republic of Korea	Retrospective	To assess two AI methods for automatically classifying dental implant diameter and length from periapical radiographs.	1320 images from 927 periapical radiographs and 874 patients	Deep learning and clustering analysis	Deep learning: VGG16 Clustering analysis: k-means++ algorithm	Both AI models demonstrated reliable classification performance.
Sakai, T. et al. (2023) [27] Japan	Retrospective	Develop an AI model to create a suitable implant drilling protocol from CBCT images.	1200	Convolutional neural network	LeNet-5	The AI model effectively predicts drilling protocols from CBCT images before surgery, suggesting it could support decision-making for achieving primary stability.
Alsomali, M. et al. (2022) [13] Saudi Arabia	Retrospective	To develop an AI model that automatically locates radiographic stent gutta percha markers in CBCT images to identify implant sites for treatment planning.	34 CBCT cases, 16,272 images	Deep learning neural network	Mask R-CNN	Training an AI program with only axial images is insufficient for accurate performance.
Lyakhov, P.A. et al. (2022) [28] Russia	Descriptive	To predict single implant survival rates using artificial intelligence.	1646 patient histories (91.64% successful implant cases (1490), 8.36% rejection cases (136)	Convolutional neural network (CNN)	PyTorch machine learning framework	The proposed neural network system offers higher accuracy than similar systems by analyzing patient data. It can only predict single dental implant outcomes and cannot be considered a complete decision support tool.
Al-Sarem et al. (2022) [25] Saudi Arabia	Retrospective	To create and analyze a deep learning model that detects missing teeth positions from segmented CBCT images.	500 CBCT images	Convolutional neural network (CNN) models	AlexNet, VGG16, VGG19, ResNet50, DenseNet169, and MobileNetV3	Among the proposed DL models, DenseNet169 performed best and can be considered a promising, time-saving tool for automated dental implant planning.
Kim et al. (2022) [22] Republic of Korea	Retrospective	To assess transfer learning in a deep convolutional neural network for classifying implant fixtures.	263 periapical radiographs with 355 implant fixtures	Deep convolutional neural network	YOLOv3	Transfer learning with YOLOv3 enabled high performance even with a small amount of data.
Sukegawa, et al. (2022) [23] Yemen	Retrospective	To evaluate the performance of the attention branch network (ABN) for implant classification using convolutional neural networks (CNNs).	10,191 dental implant images from digital panoramic radiographs (of 13 implant brands)	Convolutional neural network	ABN, ResNet 18,50,152	ResNet18 with the ABN model reported excellent performance and high compatibility in dental implant classification.
Altan et al. (2022) [26] Turkey	Retrospective	To develop and evaluate CNN (convolutional neural network) to automatically detect prosthetic restorations in panoramic radiographs using deep learning.	5126 panoramic radiographs (2988 crowns and 2969 bridges)	Convolutional neural network	YOLOv4 model	Prosthetic restorations were detected with high accuracy using the deep learning method.

**Table 2:** Quality assessment and risk of bias by the Newcastle–Ottawa Scale (NOS) for nonrandomized trials related to applications of AI in prosthodontics and implant dentistry. In the NOS scoring system, each criterion is awarded one “*” with higher overall scores corresponding to lower risk of bias.

Study	Selection				Comparability		Outcome			
	Representativeness of the Exposed Cohort	Selection of the Nonexposed Cohort	Ascertainment of Exposure	Demonstration That Outcome of Interest Was Not Present at Start of Study	Control for Main Factor	Control for Additional Factor	Assessment of Outcome	Follow-up Long Enough for Outcomes to Occur	Adequacy of Follow-up of Cohorts	Total Scores
Kurtulus et al. [14]	0	*	*	*	*	0	*	0	0	5
Chau, R.C.W. et al. [29]	0	*	*	*	*	0	*	0	0	5
Wang et al. [15]	0	*	*	*	*	0	*	0	0	5
Al Asali M. et al. [16]	0	*	*	*	*	0	*	0	0	5
Kong, H.J. et al. [12]	0	*	*	*	*	0	*	0	0	5
Seok Oh et al. [18]	*	*	*	*	*	0	*	0	0	6
Park et al. [20]	*	*	*	*	*	0	*	0	0	6
Fontenele, C.R. et al. [17]	*	0	*	*	*	0	*	0	0	5
Kong, H.J. et al. [24]	*	*	*	*	*	0	*	0	0	6
Moufti, M.A. et al. [19]	*	*	*	*	*	0	*	0	0	6
Park J H. et al. [21]	0	*	*	*	*	0	*	0	0	5
Sakai, T. et al. [27]	0	*	*	*	*	0	*	0	0	5
Alsomali, M. et al. [13]	0	*	*	*	*	0	*	0	0	5
Lyakhov, P.A. et al. [28]	0	*	*	*	*	0	*	0	0	5
Al-Sarem et al. [25]	0	0	*	*	*	0	*	0	0	4
Kim et al. [22]	0	*	*	*	*	0	*	0	0	5
Sukegawa et al. [23]	0	*	*	*	*	0	*	0	0	5
Altan et al. [26]	0	*	*	*	*	*	*	*	*	8

**Table 3:** Summaries of included studies related to ethical issues associated with the use of AI in dentistry.

Author, Year, and Country	Type of Study	Objective	Sample Size	AI Used in Study	Conclusion	Ethical Issue Discussed
Hamd, Z.Y. et al. (2023), Saudi Arabia [31]	Cross-sectional study	Willingness, knowledge, and attitude of dental professionals and students regarding use of AI in dentistry	134	NA	85.5% of dental professionals agreed on the importance of the role of AI in their practice while 31.3% stated that AI would threaten/disrupt their profession.	Professionals concerned using AI in clinical practice fearing error
Ayad, N. et al. (2023), Germany [32]	Observational study	Patient’s perception of AI in dentistry	265	DentalXrai Pro	52.5% of the population had average or above-average knowledge of AI and 47.5% had no or below-average knowledge. But their overall perception was positive toward AI use in dentistry.	Patients concerned that AI may affect workforce need and trust between dentist and patient
Roganovi’c J et al. (2023) Serbia [33]	Cross-sectional survey	Dentist’s and final-year student’s familiarity and attitude toward AI use in dentistry	193	NA	The dentists as well as the students were skeptical about AI use. Students were more anxious fearing that AI could replace dentists and also regarding the lack of regulatory policies.	Dental professionals fearing AI replacing them in the future
Rokhshad, R. et al. (2023) Multiple countries [34]	Cross-sectional	To identify the ethical challenges in using AI for smile designing	28	NA	53.6% of them had used smile designing software routinely or sometimes, 10.7% of them had used it rarely, and 35.7% had never used it.	Patient wellness, respect for autonomy, privacy protection, solidarity, governance, equity, diversity, expertise/prudence, accountability/responsibility, sustainability, and transparency
Kosan, E. et al. (2022), Germany [30]	Observational study	Patient’s perception of use of AI dentistry	140	DentalXrai	Patients showed positive perception toward use of AI in dentistry. They were able to understand what the dentist tried to explain more clearly with the help of AI.	Patients’ concern and fear about AI

**Table 4:** Quality assessment and risk of bias by the Newcastle–Ottawa Scale (NOS) for nonrandomized trials related to ethical issues associated with the use of AI in dentistry. In the NOS scoring system, each criterion is awarded one “*” with higher overall scores corresponding to lower risk of bias.

Study	Selection				Comparability		Outcome			
	Representativeness of the Exposed Cohort	Selection of the Nonexposed Cohort	Ascertainment of Exposure	Demonstration That Outcome of Interest Was Not Present at Start of Study	Control for Main Factor	Control for Additional Factor	Assessment of Outcome	Follow-up Long Enough for Outcomes to Occur	Adequacy of Follow-up of Cohorts	Total Scores
Hamd, Z.Y. et al. [31]	0	*	*	*	0	0	*	0	0	4
Ayad, N. et al. [32]	0	*	*	*	0	0	*	0	0	4
Roganovic, J. et al. [33]	0	*	*	*	0	0	*	0	0	4
Rokhshad, R. et al. [34]	0	*	*	*	0	0	*	0	0	4
Kosan, E. et al. [30]	0	*	*	*	*	0	*	0	0	5

**Table 5:** AI technologies used in prosthodontics and implant dentistry.

AI Technology	Description	Applications
Convolutional Neural Networks (CNNs)	Deep learning models specialized for image analysis, particularly in feature extraction and classification.	Classification of dental radiographs, implant system identification, and bone analysis.
Generative Adversarial Networks (GANs)	AI models designed for generating new data that mimics existing datasets.	Designing dental prostheses and simulating natural tooth morphology.
U-Net	A neural network architecture for image segmentation, especially for medical imaging tasks.	Tooth segmentation, bone segmentation, and implant site assessment from CBCT or 3D scans.
YOLO (You Only Look Once)	Object detection algorithm optimized for real-time processing and detection.	Detection of dental prosthetics, restorations, and implant fixtures in radiographic images.
ResNet (Residual Networks)	Deep learning architecture designed to overcome vanishing gradient problems in deep neural networks.	Osseointegration prediction and implant classification.
DenseNet (Dense Convolutional Networks)	AI models focus on efficient parameter sharing to improve performance with fewer computations.	Prediction of implant outcomes and bone density assessment.
Support Vector Machines (SVMs)	A supervised learning model for classification and regression analysis.	Classifying dental materials and differentiating between healthy and diseased tissue.
Random Forests	Ensemble learning techniques that combine multiple decision trees for classification and regression.	Predicting implant success rates and analyzing patient-specific prosthodontic outcomes.
Attention Mechanisms (e.g., Attention Branch Network)	Models that focus computational resources on relevant parts of input data for improved accuracy.	Dental implant classification and anomaly detection in imaging data.
3D Neural Networks	Extensions of CNNs for analyzing volumetric data.	3D segmentation of bone structures, implant planning, and prosthesis fitting.

## Data Availability

The data are contained within the article.
